# Meta-Analysis of Contemporary Trials of Omega-3 Fatty Acids Containing Both Eicosapentaenoic and Docosahexaenoic Acids

**DOI:** 10.1016/j.eclinm.2021.101110

**Published:** 2021-08-25

**Authors:** Safi U. Khan, Deepak L. Bhatt

**Affiliations:** a Department of Cardiology, Houston Methodist DeBakey Heart & Vascular Center, Houston TX; b Brigham and Women's Hospital Heart and Vascular Center, Harvard Medical School, Boston, MA

Drs. Mason and Eckel raise the concern that older trials of combined eicosapentaenoic acid (EPA) and docosahexaenoic acid (DHA) with suboptimal statin therapy can bias results in favor of EPA+DHA. The authors correctly pointed out that GISSI-P [1] and GISSI-HF [2] were notably different from contemporary trials in the use of background statin therapy even at the end of the trials. Our meta-analysis followed a pre-specified study selection criteria and included all the eligible studies to avoid selection and reporting biases [3]. However, we agree with the authors that both the GISSI-P [1] and GISSI-HF [2] trials (42% relative weight in the EPA+DHA meta-analysis) carry considerable potential to influence results in favor of EPA+DHA therapy. Therefore, we repeated the meta-analysis of EPA+DHA trials excluding these older trials [1, 2] and found that EPA+DHA therapy was associated with neither lower cardiovascular mortality (RR: 0.96 [0.90-1.03]; *P* = 0.30) nor reduced non-fatal cardiovascular outcomes (Figure).

**Figure:**
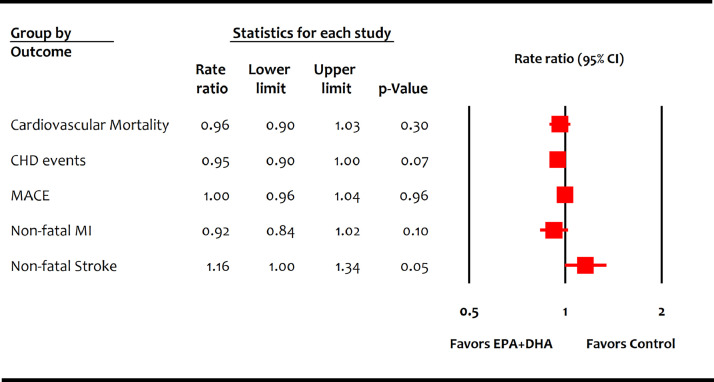
Meta-analysis of contemporary trials of omega-3 fatty acids containing mixed formulations of eicosapentaenoic acid (EPA) and docosahexaenoic acid (DHA). CI = confidence interval; CHD = coronary heart disease; MI = myocardial infarction; MACE = major adverse cardiovascular events.

Our meta-analysis [3] concluded that cardiovascular risk reduction was present with EPA monotherapy more so than with combined EPA+DHA. We thank Drs. Mason and Eckel for their insightful observation that allowed us to clarify further that the cardiovascular benefits of omega-3 FAs in the contemporary era are limited to EPA monotherapy. These findings align with recent trials showing remarkable cardiovascular risk reduction with EPA monotherapy [4, 5] and underscore that the beneficial effects of prescription EPA should not be generalized to mixed prescription formulations of omega-3 FAs or poorly regulated dietary supplements.

## Author Contributions

Safi U. Khan: data abstraction, analysis, and manuscript drafting.

Deepak L. Bhatt: supervision and critical revision.

## Declaration of Competing Interest

Dr. Safi Khan has nothing to disclose.

Dr. Bhatt reports grants from Amarin, grants from AstraZeneca, grants from Bristol-Myers Squibb, grants from Eisai, grants from Ethicon, grants from Medtronic, grants from sanofi aventis, grants from The Medicines Company, other from FlowCo, grants and other from PLx Pharma, other from Takeda, personal fees from Duke Clinical Research Institute, personal fees from Mayo Clinic, personal fees from Population Health Research Institute, personal fees, non-financial support and other from American College of Cardiology, personal fees from Belvoir Publications, personal fees from Slack Publications, personal fees from WebMD, personal fees from Elsevier, other from Medscape Cardiology, other from Regado Biosciences, other from Boston VA Research Institute, personal fees and non-financial support from Society of Cardiovascular Patient Care, non-financial support from American Heart Association, personal fees from HMP Global, grants from Roche, personal fees from Harvard Clinical Research Institute (now Baim Institute for Clinical Research), other from Clinical Cardiology, personal fees from Journal of the American College of Cardiology, other from VA, grants from Pfizer, grants from Forest Laboratories/AstraZeneca, grants from Ischemix, other from St. Jude Medical (now Abbott), other from Biotronik, grants and other from Cardax, other from Boston Scientific, grants from Amgen, grants from Lilly, grants from Chiesi, grants from Ironwood, personal fees from Cleveland Clinic, personal fees from Mount Sinai School of Medicine, other from Merck, grants from Abbott, grants from Regeneron, other from Svelte, grants and other from PhaseBio, grants from Idorsia, grants from Synaptic, personal fees from TobeSoft, grants, personal fees and other from Boehringer Ingelheim, personal fees from Bayer, grants and other from Novo Nordisk, grants from Fractyl, personal fees from Medtelligence/ReachMD, personal fees from CSL Behring, grants and other from Cereno Scientific, grants from Afimmune, grants from Ferring Pharmaceuticals, other from CSI, grants from Lexicon, personal fees from MJH Life Sciences, personal fees from Level Ex, grants from Contego Medical, grants and other from CellProthera, personal fees from K2P, personal fees from Canadian Medical and Surgical Knowledge Translation Research Group, grants and other from MyoKardia/BMS, grants from Owkin, grants from HLS Therapeutics, grants and other from Janssen, grants from 89Bio, grants and other from Novo Nordisk, grants from Garmin, grants and other from Novartis, grants and other from NirvaMed, other from Philips, outside the submitted work.
